# *Gingko biloba* Extract (EGb) Inhibits Oxidative Stress in Neuro 2A Cells Overexpressing APPsw

**DOI:** 10.1155/2019/7034983

**Published:** 2019-07-11

**Authors:** Le Chen, Chenghong Zhang, Ying Han, Xianyi Meng, Ying Zhang, Haiying Chu, Haiying Ma

**Affiliations:** Department of Histology and Embryology, College of Basic Medical Sciences, Dalian Medical University, 116044 Dalian, Liaoning, China

## Abstract

Alzheimer's disease (AD) is a common neurodegenerative disease. Abundant evidence demonstrates that oxidative stress may be not only an early event in this disease, but also a key factor in the pathogenesis of AD.* Ginkgo biloba* extract (EGb) has a strong ability to scavenge oxygen free radicals and supply hydrogen. The present study aims to investigate the effects of EGb on Neuro 2A cells transfected with Swedish mutant APP (APPsw). Stably transfected Neuro 2A cell lines expressing human wild-type APP (APP695), APPsw, or empty vector(neo) pEGFP-N2 were treated with 100 *μ*g/ml EGb for 0, 2, 4, 6, 8, and 10 h. Oxidative stress was assessed by measuring free radicals and the activities of antioxidant enzymes. Our studies showed that EGb treatment reduced the production of reactive oxygen species (ROS) and the levels of malondialdehyde (MDA) significantly while total superoxide dismutase (T-SOD), catalase (CAT), and glutathione peroxidase (GSH-Px) activities were enhanced in Neuro 2A cells overexpressing APPsw. Meanwhile, A*β* levels in these cells were also reduced compared to the levels in untreated cells and control cells (empty vector(neo) pEGFP-N2). These findings suggest that EGb can reduce oxidative stress by decreasing free radical and enhancing antioxidant status, further leading to reduced A*β* aggregation; EGb might be a potential therapeutic agent for Alzheimer's disease (AD).

## 1. Introduction

Alzheimer's disease (AD) is a neurodegenerative disease with progressive cognitive dysfunction and memory impairment and is the main cause of dementia in elderly people over 60 years old. AD is characterized by neuronal loss, extracellular deposits of *β*-amyloid (A*β*) in senile plaques and intraneuronal neurofibrillary tangles [1, 2]. According to the “A*β* cascade hypothesis” mechanism, A*β* is a key protein in AD pathology that is derived from the processing of transmembrane amyloid precursor protein (APP) by different secretases.

A large body of evidence has shown that free radicals may be involved in the etiopathogenesis of AD, and oxidative stress in neurons precedes and accompanies the accumulation of A*β* in AD [3–8]. Oxidative stress increases *β*- and *γ*-secretase activity and promotes the accumulation and deposition of A*β*. On the other hand, A*β* can promote the occurrence of oxidative stress, eventually forming a vicious circle and causing irreversible oxidative damage [9–11]. In addition, it is believed that oxidative damage to critical molecules occurs early in the pathogenesis of AD and precedes pronounced neuropathological alterations [12]. Because oxidative damage begins early in the progression of the disease, it represents a potential therapeutic target for slowing the onset and progression of AD [13].

*Ginkgo biloba* extract (EGb) is derived from the leaves of* Ginkgo biloba* and its main active constituents are flavonoid glycosides, bilobalide, and ginkgolides. EGb can remove excessive oxygen free radicals, inhibit lipid peroxidation of cell membranes, inhibit inflammation and allergic reactions, and modulate immune responses, and it also has a strong hydrogen supply capacity [14]. Many studies have shown that the effects of EGb involve its antioxidant properties [15, 16].

Therefore, in the current study, Neuro 2A (N2a) cells transfected with human APP695 and its Swedish mutant APPsw were used as an* in vitro* model of AD. We investigated the effect of EGb on this AD model. We examined cellular eactive oxygen species (ROS) production and assessed changes in intracellular malondialdehyde (MDA), total superoxide dismutase (T-SOD), catalase (CAT), and glutathione peroxidase (GSH-Px). The findings indicated that EGb showed significant antioxidant effects in N2a-APPsw cells, which may delay the onset and progression of AD.

## 2. Materials and Methods

### 2.1. Cell Culture, Transfection, and Treatment

N2a cells were cultured in MEM-GlutaMAX media (HyClone, USA) supplemented with 10% FBS (BI), 100 U/ml penicillin, and 100 *μ*g/ml streptomycin in a 5% CO_2_ humidified atmosphere at 37°C. Stably transfected N2a cell lines expressing human APP695 and APPsw or the empty vector(neo) pEGFP-N2 were established by using the Effectene Transfection Reagent (QIAGEN, Germany) and selected by G418 resistance.

Cells were treated with 0, 50, 100, 200, or 400 *μ*g/ml EGb (final concentration) for 8 h for the CCK-8 assay; treated with 100 *μ*g/ml EGb for 0, 2, 4, 6, 8, or 10 h for the ROS assay; and treated with 100 *μ*g/ml EGb for 8 h for the MDA, T-SOD, CAT, and GSH-Px assays. Cells in the control group were treated with DMEM medium alone as the vehicle.

### 2.2. CCK-8 Assay

Cell viability was measured in 96-well plates by CCK-8 assay. Briefly, after cells were treated with vehicle or EGb for the indicated time, 100 *μ*l CCK-8 was added to the medium and then incubation continued at 37°C for 1 h. The absorbance at 450 nm was measured using a microplate reader (Thermo Scientific). The cell viability was expressed as the ratio of the signal obtained from the treated group to the control group.

### 2.3. Western Blot Assay

The cells were lysed on ice with precooled lysis buffer. After centrifugation at 12,000* rpm* for 15 min at 4°C, the total protein concentration of each experimental group was quantified using a BCA kit (Beyotime Biotechnology, China). Equivalent protein lysates (30 *μ*g) were separated by 10% SDS-PAGE gels and blotted onto PVDF membranes (Millipore, USA). Proteins were detected by using antibodies anti-APP (1:1000, Sigma, USA) and anti-*β*-actin (1:1000, Santa Cruze, USA) overnight at 4°C. After incubation with the secondary antibody, signals were visualized using an enhanced chemiluminescence detection kit (Advansta, USA) and quantified using Quantitative One Image Analysis (BioRad, USA).

### 2.4. Immunofluorescence Staining

Cells were seeded in 12-well slides (Solarbio, China) and treated as described above. The slides were washed with ice-cold PBS, fixed with 4% PFA at room temperature for 15 min, washed 3 times with PBS, and incubated with a 5% BSA blocking solution at room temperature for 2 h. The slides were incubated with rabbit anti-A*β* (1:200, Cell Signaling Technology) antibody overnight at 4°C. After washing with PBS, and the sample were incubated with Alexa Fluor-488 conjugated goat anti-rabbit antibody (1:300, Vector laboratories, USA) at room temperature for 2 h. Cells were examined using a fluorescence microscope (Olympus, Japan), and the mean optical density was quantified using Image-pro plus 5.1 software.

### 2.5. Oxidative Stress Assays: MDA, T-SOD, CAT, and GSH-Px Content Measurement

Cells cultured for 24 h in 6-well plates (4 × 10^4^ cells/well) were treated with vehicle or 100 *μ*g/ml EGb for 8 h. Cells were digested with trypsin and centrifuged at 1000 rpm for 5 min. Thereafter, the cells were suspended in 500 *μ*l PBS, and lysed by sonication in the presence of a protease inhibitor, followed by centrifugation at 4000 rpm for 15 min. The supernatant was collected for analysis, according to the assay kits (JianCheng Biology, China) manufacturer's instructions. MDA absorbance was recorded at 532 nm, and the results were calculated and expressed as nmol/mg protein. T-SOD, CAT, and GSH-Px in the cell homogenates were determined by colorimetry analysis at 550, 405, and 412 nm, respectively. The activities of these enzymes are expressed as U/mg protein.

### 2.6. ROS Production

Intracellular ROS levels were measured using the redox-sensitive fluorescent dye, DCFH-DA from ROS assay kits (Beyotime, China). Conversion of nonfluorescent DCFH-DA to fluorescent dichlorofluorescein (DCF) in the presence of ROS was measured on a microplate reader. In brief, cells were seeded in 96-well plates at a density of 2 × 10 ^4^ cells per well. Following drug treatment, cells were washed three times with PBS, incubated with 10 *μ*M DCFH-DA for 30 min at 37°C in the dark, and washed three times with PBS to remove the extracellular DCFH-DA. The fluorescence emission intensity of DCF (525 nm) was measured in response to 488 nm excitation. The level of intracellular ROS was expressed as the percentage of control cultures incubated in DCFH-DA and the quantification of mean optical density was analyzed by Image-pro plus 5.1 software.

### 2.7. Statistical Analysis

All values were expressed as the mean ± standard deviation (SD). The statistical analyses were then completed with one-way analysis of variance (ANOVA) and Student's t-test. Differences were considered significant at* p* < 0.05.

## 3. Results

### 3.1. A Stable Cell Model Overexpressing Human APP695 (N2a-APP695) and APPsw (N2a-APPswe) Was Established

**Figure 1:**
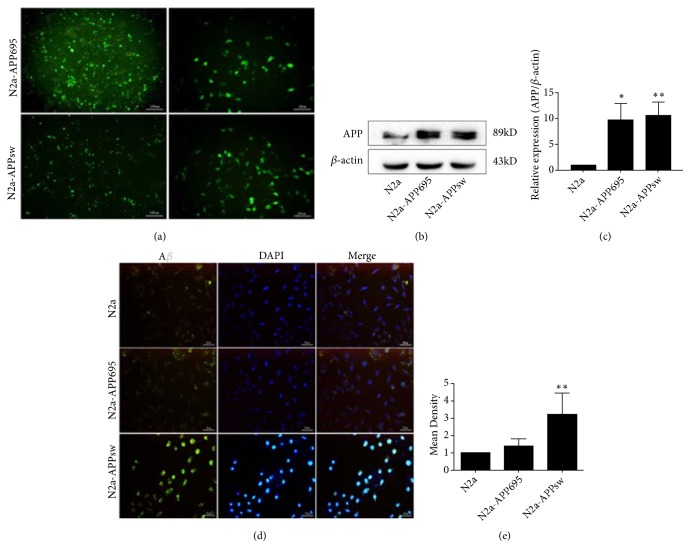
A stable overexpressing human APP695 (N2a-APP695) and APPsw (N2a-APPswe) cell model. (a) Green fluorescent protein can be observed in transfected Neuro 2A cells. (b), (c) The expression of human APP was detected by Western blot. Compared with N2a cells, the expression of APP in N2a-APP695 and N2a-APPsw was significantly increased. (d), (e) The expression of human A*β* was detected by immunocytochemical staining. Compared with control cells, A*β* was significantly increased in N2a-APPsw cells. All values are presented as the mean ± SD from three independent experiments. *∗p*<0.05, *∗∗p*<0.01.

To resemble APP expression and A*β* secretion, a N2a cell line overexpressing the human APP Swedish mutant was developed (Figure 1). Green fluorescence can be observed in transfected cells (a). The APP protein revealed a major band at 86 kDa (b), and its expression was significantly increased in N2a-APP695 (*p*<0.05) and N2a-APPsw cells (*p*<0.01) compared with N2a cells (c). Immunofluorescence intensity was performed to assess the A*β* level, and N2a-APPsw cells exhibited an increased A*β* level compared with N2a (*p*<0.01) and N2a-APP695 cells (*p*<0.05) (d & e).

### 3.2. EGb Optimal Concentration Screening

**Figure 2:**
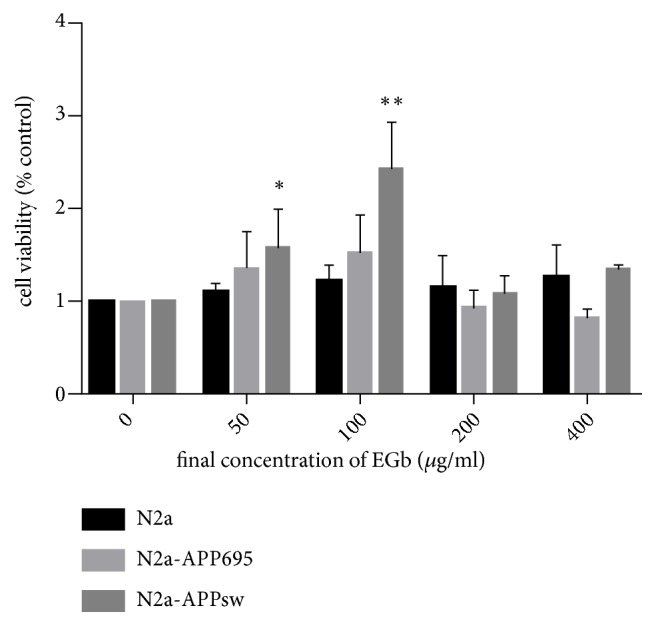
The effect of EGb on N2a, N2a-APP695, and N2a-APPsw cell viability. The viability of N2a, N2a-APP695, and N2a-APPsw cells was measured by CCK-8 assay at different concentrations of EGb (50, 100, 200, or 400 *μ*g/ml) for 8 h. Cells cultured in 100 *μ*g/ml EGb exhibited the highest viability (*p*<0.01). All values are presented as the mean ± SD from three independent experiments. *∗p*<0.05, *∗∗p*<0.01.

To evaluate the effect of EGb on the viability of N2a-APPsw cells, cells were treated with different doses of EGb (50, 100, 200, or 400 *μ*g/ml) for 8 h (Figure 2). The data showed that cells cultured in 100 *μ*g/ml EGb exhibited the highest cell viability (*p*<0.01); thus, in the following experiments for the determination of survival in response to different treatments, the optimum concentration of EGb was 100 *μ*g/ml. Cells in the control group were treated with vehicle alone.

### 3.3. EGb Reduced ROS Production in N2A-APPsw Cells

**Figure 3:**
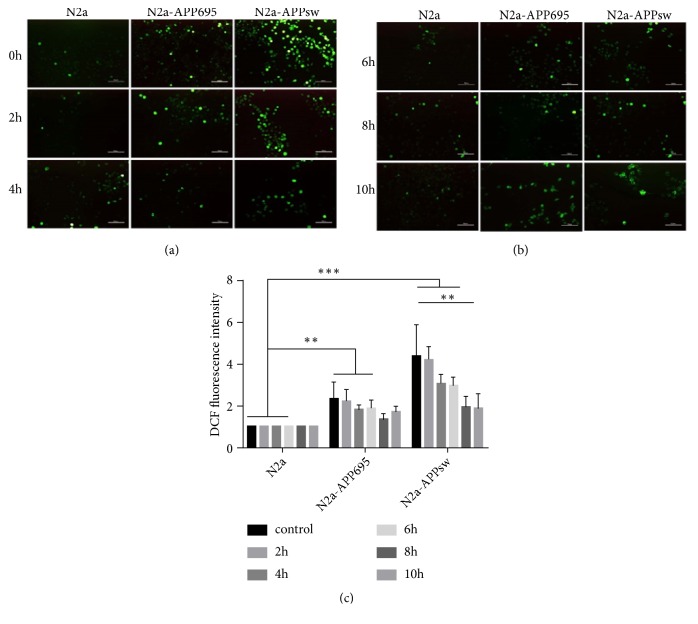
The effects of EGb on intracellular ROS in N2a, N2a-APP695, and N2a-APPsw cells. (a), (b) Intracellular ROS was detected by DCFH-DA in N2a, N2a-APP695, and N2a-APPsw cells. Fluorescence intensity represents ROS level. (c) Fluorescence intensity was evaluated. n = 5 in each group. All values are presented as the mean ± SD from three independent experiments. *∗p* <0.05, *∗∗p*<0.01, and *∗∗∗p*< 0.001.

The relative fluorescence intensity of ROS in N2a cells without any treatment was chosen as the stanard. ROS levels were significantly increased in N2a-APPsw cells (*p*<0.001). EGb was significantly reduced ROS levels. As the drug intervention time increased, ROS levels continued to decrease in cells. At 8 h, the effect of the drug was clear: the production of ROS in the cells was significantly decreased (*p*<0.05), and there was no significant difference compared with the control group (Figure 3).

### 3.4. EGb Reduced MDA Production in N2a-APPsw Cells

**Figure 4:**
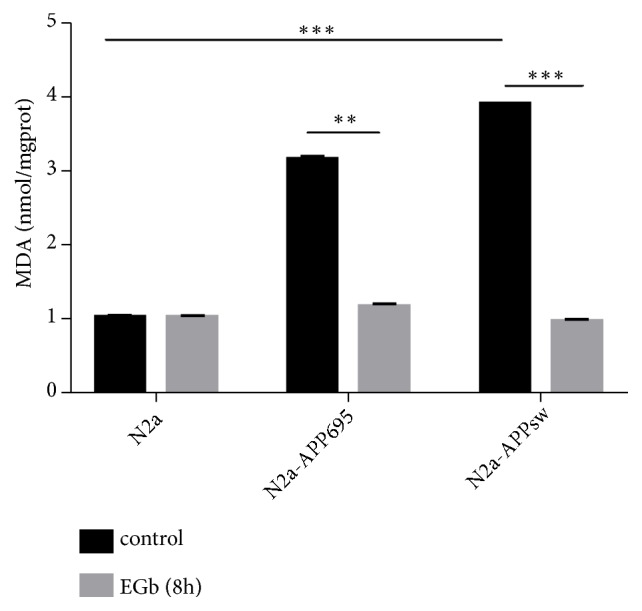
Effects of EGb on the activity an oxidative product in N2a, N2a-APP695, and N2a-APPsw cells. N2a, N2a-APP695, and N2a-APPsw cells were treated with 100 *μ*g/ml EGb for 0 or 8 h. Compared with N2a cells, the activity of MDA in N2a/APPsw cells was significantly increased (*p*<0.001). After treatment with 100 *μ*g/ml EGb, the activity of MDA in cell homogenates was significantly decreased (*p*<0.001). All values are presented as the mean ± SD from three independent experiments. *∗p* <0.05, *∗∗ p* <0.01, and *∗∗∗ p* <0.001.

The concentration of MDA is an indicator of oxidative stress. As shown in Figure 4, intracellular MDA concentrations were significantly increased in N2a-APP695 and N2a-APPsw cells compared with the negative control group cells (*p*<0.001). However, after treatment with 100 *μ*g/ml EGb, the activity of MDA in cell homogenates was significantly decreased compared with the activity before drug treatment (*p*<0.01).

### 3.5. EGb Increased T-SOD, CAT, and GSH-Px Levels in N2a-APPsw Cells

**Figure 5:**
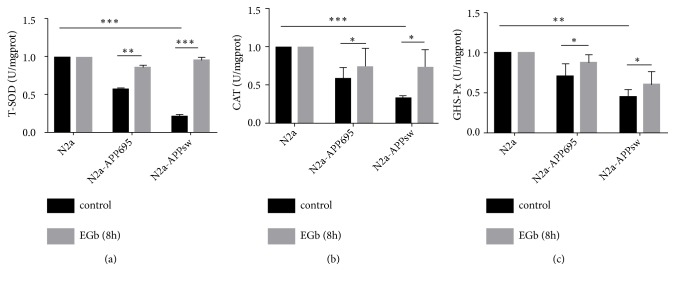
Effects of EGb on antioxidant enzyme activities in N2a, N2a-APP695, and N2a-APPsw cells. Compared with N2a cells, the activity of T-SOD (a), CAT (b), and GSH-Px (c) in N2a-APP695 and N2a-APPsw cells was significantly decreased. However, after treatment with 100 *μ*g/ml EGb for 8 h, the activities of the antioxidant enzymes were significantly increased in the cell homogenates from N2a-APP695 and N2a-APPsw. All values are presented as the mean ± SD from three independent experiments. *∗p* <0.05, *∗∗ p* <0.01, and *∗∗∗ p* <0.001.

As shown in Figure 5, intracellular T-SOD, CAT, and GSH-Px concentrations were significantly decreased in N2a-APP695 and N2a-APPsw cells compared with the concentrations in control group cells (T-SOD,* p*<0.001; CAT,* p*<0.05; GSH-Px,* p*<0.05). After treatment with 100 *μ*g/ml EGb, the activities of T-SOD, CAT, and GSH-Px in N2a-APPsw cell homogenates were significantly increased compared with the activities before drug treatment (T-SOD,* p*<0.001; CAT,* p*<0.05; GSH-Px,* p*<0.01).

### 3.6. EGb Decreased the Expression of A*β* in N2a-APPsw Cells

**Figure 6:**
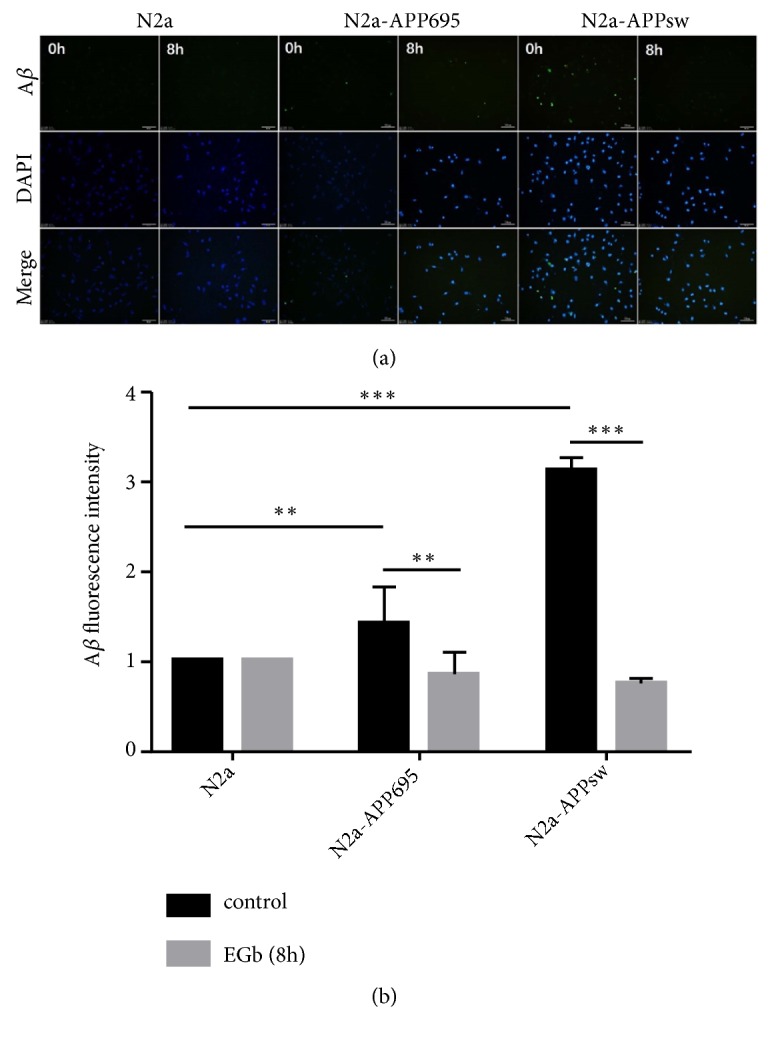
EGb decreased the expression of A*β* in N2a-APPsw cells. (a) Intracellular A*β* expression was detected by immunofluorescence. (b) The expression of A*β* in N2a-APPsw cells was significantly increased (*p*<0.001). After treatment with 100 *μ*g/ml EGb for 8 h, the expression of A*β* was decreased (*p*<0.001). All values are presented as the mean ± SD from three independent experiments. *∗p*<0.05, *∗∗ p* <0.01, and *∗∗∗ p* <0.001.

In addition to plaques, the excessive accumulation of A*β* can also lead to mitochondrial dysfunction, such as mitochondrial depolarization and oxidative stress. Thus, we examined the effect of EGb on the expression of A*β* in N2a, N2a-APP695, and N2a-APPsw cells. As shown by the immunofluorescence results shown in (Figure 6) (a), the expression of A*β* in N2a-APPsw cells was significantly increased (*p*<0.001). As expected, EGb was able to reduce intracellular A*β* compared with the control group (*p *<0.01) (b).

## 4. Discussion

A*β* peptides are considered the primary pathological agents in AD. As a precursor protein of A*β*, APP is a protein that has been widely studied in the field of AD pathology [17, 18]. APP-transfected neuron-like cells have robust expression of APP and make for a reliable system to produce A*β* production* in vitro*, which can model the pathological characteristics of AD [19]. Therefore, the N2a cell line stably overexpressing human APP695 and its Swedish mutant APPsw was used as the research model in this study. As measured by inverted fluorescence microscopy, the expression level of the APP protein and A*β* in N2a-APPsw cells indicated that the cell model was successfully constructed* in vitro* and could be used in subsequent experiments. The advantage of this cell line is that it stabilizes the overexpression of human APP695 and its Swedish mutants, reducing individual differences between cells and better simulating the* in vivo* environment.

The currently available synthetic compounds that are applied in the treatment of neurodegenerative diseases such as AD have failed to work as initially expected and often induce various side effects [20]. Therefore, natural compounds with potentially multiple targets of neuroprotective effects derived from plants, such as curcumin, vitamin C, and* Gingko biloba*, have been investigated intensively in recent years [21].

EGb is a highly effective oxygen free radical scavenger. It can inhibit lipid peroxidation and inflammation, and its protective effect on the central nervous system is closely related to its antioxidation effect. It also is used as a vasodilator to treat ischemic conditions of the brain, renal, lung, and hear [22–25]. In addition, EGb was shown to relieve DSS-induces acute experimental colitis by promoting apoptosis [14, 25, 26]. To explore the effect of EGb on the activity of transfected APPsw cells, we treated cells with different concentrations of EGb for 8 h. We found that 100 *μ*g/ml EGb significantly increased the activity of cells. However, the activity of cells was decreased with the increased concentration. This result indicated that EGb within a dose range is protective for cell growth but beyond that can be detrimental.

Oxidative stress is also a significant pathological feature of the neurodegenerative disease, such as Parkinson's disease [27], Huntington's disease [28], and Alzheimer's disease (AD) [29, 30]. Oxidative stress is the condition that can result from an imbalance in reactive oxygen species (ROS), and that is a key mechanism of cell death. The overproduction of ROS, such as free radicals, superoxide, and hydrogen peroxide, can damage the physiological functions of cellular proteins, lipids, nucleic acids, and other macromolecules directly or indirectly [31]. In the current study, the results showed that the ROS concentration in N2a-APPsw cells was higher than the level in N2a-APP695 and N2a normal cells. However, after treatment with 100 *μ*g/ml of EGb, the ROS content was decreased, and it decreased to the lowest at 8 h in N2a-APP695 cells and N2a-APPsw cells. This demonstrated that increased A*β* expression in N2a-APPsw cells stimulates the increase of ROS. A*β* induces the production of oxygen free radicals, and proteins and lipids in the cell membrane system of the brain are oxidized [32], which increases the production of ROS. In addition, aggregation of A*β* reduces mitochondrial redox activity, resulting in the accumulation of ROS. We found that EGb reduced ROS production as well as aggregation of A*β* and had the best protective effect in N2a-APPsw cells at 8 h.

Furthermore, we also examined the concentrations of MDA. The metabolism of lipid peroxides produces MDA, indirectly reflecting the severity of free radical attack by. Consistent with previous results [33], we found that the concentration of MDA was significantly increased in N2a-APPsw cells. However, it was significantly decreased by EGb treatment.

T-SOD, CAT, and GSH-Px are the most important antioxidant enzymes that act against oxygen free radicals and regulate the metabolism of free radicals in the body and play a role in the free radical scavenging system, protecting the cells in the body from lipid peroxidation. The large amount of ROS reacts with biomacromolecules and unsaturated fatty acids to reduce the activity of antioxidant enzymes. Therefore, the activities of T-SOD, CAT, and GSH-Px reflect the ability of the body to scavenge oxygen free radicals. In this study, the concentrations of T-SOD, CAT and GSH-Px were remarkably decreased in the model cells, and EGb significantly increased the concentrations of T-SOD, CAT, and GSH-Px.

Therefore, we deduced that the concentrations of ROS, MDA, T-SOD, CAT, and GSH-Px were implicated in the development of AD and that EGb played its neuroprotective roles by increasing the expression of T-SOD, CAT, and GSH-Px or by decreasing both ROS and MDA to exert a direct free radical scavenging effect. A*β* deposition in the brain was related to the increased oxidative stress in AD patients and transgenic mouse models of AD [7, 34]. A*β* production has been shown to increase following events of oxidative stress and neuronal energy reduction [19, 35, 36]. Our results suggested that EGb can reduce oxidative stress and A*β* production, which may directly interact with one another in N2a-APP695 and N2a-APPsw cells.

*Ginkgo biloba* is currently the most widely used herb in the study of cognitive disorders and AD. However, its efficacy in preventing and treating dementia remains unclear [37]. Some research shown the value of EGb for treating AD has become increasingly negative [38]. However, it was verified that compounds contained in the EGb can cross the blood-brain barrier and inhibit the production of free radicals to cause pharmacological effects. Ginkgolides, biologically active terpenic lactones in* Ginkgo biloba*, are scavengers of reactive oxygen species and can inhibit A*β*-induced cell death [39]. The bilobalide also has strong antioxidant activity and can promote expression of growth factors and lead neural to growth [40]. Numerous studies in animals, healthy elderly humans, and AD patients indicated that EGb has shown protective properties against vascular and neuronal damage [41, 42] and the significant improvement in cognitive function [43, 44]. In our study, we also demonstrated that EGb plays an important role in combating oxidation and attenuating the expression of A*β* in N2a-APPsw cells. Thus, EGb might be a potential therapeutic agent for AD. In the next study, we will focus on the underlying mechanisms of EGb's antioxidative effects in AD.

## Data Availability

The data used to support the findings of this study are available from the corresponding author upon request.
